# Texas State Medical Association, Note from Secretary

**Published:** 1891-07

**Authors:** H. A. West

**Affiliations:** Secretary; Galveston


### Texas State Medical Association—A Word About the Transactions.

I desire to say a few words to the members and ex-members of the Asssociation in advance of the appearance of the Transactions, in the way of explanation, exhortation and apology. The Transactions will be ready for delivery early in August; at this date most of the printing is already done. I have taken the utmost pains to secure a correct list of members, in spite of which, doubtless, some errors in names and post office addresses may exist. Many, perhaps, may be surprised to find their names off the roll. My brother, for this do not blame the Secretary or the Treasurer; perhaps you have been dropped from the roll for non-payment of dues, in accordance with the constitution and by-laws. To remedy this, send your ten dollars promptly to Dr. J. Larendon, Houston, and your name will be added and the Transactions sent to you. If you find yourself in the anomalous position of an officer of the Association, when, in reality, you are not a member, set yourself right by paying your dues. (There are at least two instances where this is the case.) The Secretary has nothing whatever to do with the money matters of the Association; these duties were wisely relegated, at the Waco meeting, to the Treasurer, where they properly belong. The list of members in good standing is furnished by the Treasurer to the Secretary. Please do not labor under the erroneous impression that the treasury is plethoric with funds; on the contrary, I have been unable to furnish the superior binding I wanted, because the money to pay for the same was lacking. The constitution, by-laws and ordinances you will find at the beginning of the book. This change of position was made for a purpose. It is my belief that many of the errors and omissions of the past were due, not to any defects of the constitution, but because of an unfamiliarity with the requirements of the same. Let me kindly admonish you to read, mark, inwardly digest and observe the laws we have adopted for our government. If you have changed your residence, please notify me promptly. The discussions, as reported by the stenographer, were found to be very imperfect, due partly to the reporter's non-acquaintance with technical terms, the difficulty of hearing in the hall, and (I can say it as I was one of the number) to the repetitions and careless methods of the speakers themselves. While it has been found necessary to alter the verbiage, I have endeavored to give the sense of what was said. As to the imperfections and omissions you will doubtless find in the coming volume, bear in mind I am new to this work, and cover them with the "mantle of charity." Fraternally yours,

H. A. WEST, Secretary.

GALVESTON, July 17, 1891.

